# CNS-Related Effects Caused by Vanadium at Realistic Exposure Levels in Humans: A Comprehensive Overview Supplemented with Selected Animal Studies

**DOI:** 10.3390/ijms24109004

**Published:** 2023-05-19

**Authors:** Agnieszka Ścibior, Juan Llopis, Paweł Piotr Dobrakowski, Tomasz Męcik-Kronenberg

**Affiliations:** 1 Laboratory of Oxidative Stress, Department of Biomedicine and Environmental Research, Institute of Biological Sciences, Faculty of Medicine, The John Paul II Catholic University of Lublin, Konstantynów St. 1J, 20-708 Lublin, Poland; 2 Department of Physiology, Institute of Nutrition and Food Technology ’’José Mataix“, Biomedical Research Centre, University of Granada, 18100 Armilla, Spain; jllopis@ugr.es; 3 Sport and Health Research Centre, University of Granada, 18016 Granada, Spain; 4 Psychology Institute, Humanitas University in Sosnowiec, Jana Kilińskiego St. 43, 41-200 Sosnowiec, Poland; paweldobrakowski@interia.pl; 5 Department of Pathomorphology, Faculty of Medical Sciences in Zabrze, Medical University of Silesia, 3 Maja St. 13, 41-800 Zabrze, Poland; patolog@interia.pl

**Keywords:** vanadium, neurodegenerative diseases, neurological/neurobehavioral effects, environmental/occupational exposure, humans, body fluids, brain

## Abstract

Neurodegenerative disorders, which are currently incurable diseases of the nervous system, are a constantly growing social concern. They are progressive and lead to gradual degeneration and/or death of nerve cells, resulting in cognitive deterioration or impaired motor functions. New therapies that would ensure better treatment results and contribute to a significant slowdown in the progression of neurodegenerative syndromes are constantly being sought. Vanadium (V), which is an element with a wide range of impacts on the mammalian organism, is at the forefront among the different metals studied for their potential therapeutic use. On the other hand, it is a well-known environmental and occupational pollutant and can exert adverse effects on human health. As a strong pro-oxidant, it can generate oxidative stress involved in neurodegeneration. Although the detrimental effects of vanadium on the CNS are relatively well recognized, the role of this metal in the pathophysiology of various neurological disorders, at realistic exposure levels in humans, is not yet well characterized. Hence, the main goal of this review is to summarize data on the neurological side effects/neurobehavioral alterations in humans, in relation to vanadium exposure, with the focus on the levels of this metal in biological fluids/brain tissues of subjects with some neurodegenerative syndromes. Data collected in the present review indicate that vanadium cannot be excluded as a factor playing a pivotal role in the etiopathogenesis of neurodegenerative illnesses, and point to the need for additional extensive epidemiological studies that will provide more evidence supporting the relationship between vanadium exposure and neurodegeneration in humans. Simultaneously, the reviewed data, clearly showing the environmental impact of vanadium on health, suggest that more attention should be paid to chronic diseases related to vanadium and to the assessment of the dose–response relationship.

## 1. Introduction

**Figure 1:**
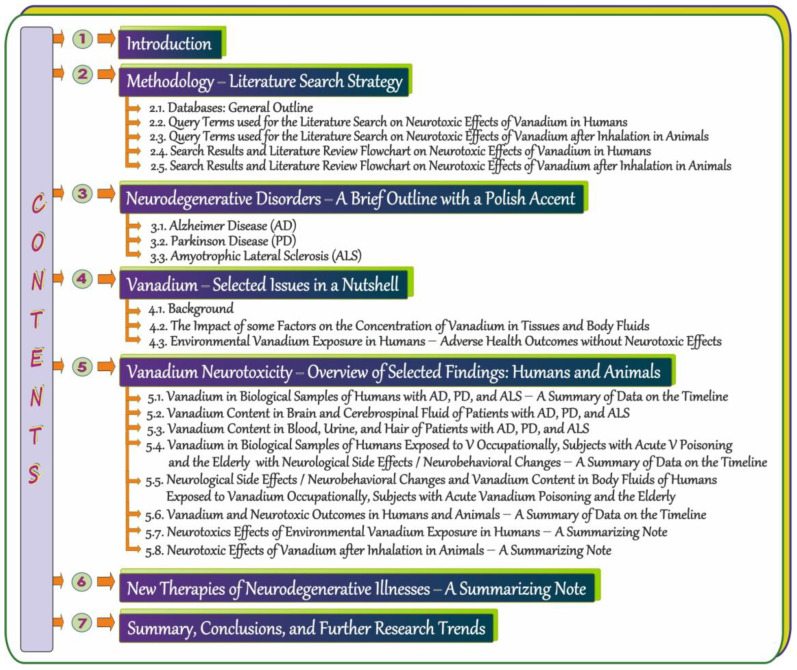
Graphical summary of the overviewed issues.

The present review is an attempt to provide thorough knowledge on the influence of vanadium (V) on neurodegeneration in humans. After the Introduction section, in which the main goals are formulated, the review comprises a few main sections and subsections displayed in Figure 1 for the reader’s convenience. Section 2 provides information about the search strategy, i.e., it includes the sources and date of searching, the key terms used to identify records relevant to the topic, and two flowcharts of the literature review process. One of them refers to humans and the other one is focused on animals. Section 3 with a Polish accent provides a concise summary of basic information about certain neurogenerative diseases, i.e., Alzheimer disease (AD), Parkinson disease (PD), and Amyotrophic Lateral Sclerosis (ALS). Section 4, composed of three Section 4.1, Section 4.2 and Section 4.3 overviews, highlight selected issues on vanadium. The first subsection presents a brief background related to this metal, the second one summarizes data on the impact of some factors on the concentration of vanadium in tissues and body fluids, and the last one collects information about adverse health outcomes of environmental exposure to vanadium in humans with the exception of neurotoxic effects. Section 5 with eight Section 5.1, Section 5.2, Section 5.3, Section 5.4, Section 5.5, Section 5.6, Section 5.7 and Section 5.8 compiles data on the content of vanadium in biological specimens from patients with AD, PD, and ALS, summarizes the neurotoxic effects of environmental exposure to this metal in humans, overviews findings from studies on the neurotoxic effects of vanadium after inhalation in animals (in both laboratory animals and those naturally exposed to urban air), and collects information about the neurological side effects and neurobehavioral changes in humans exposed to vanadium occupationally, subjects with acute vanadium poisoning, and the elderly. Some of these, Section 5.1, Section 5.4 and Section 5.6, illustrate selected issues related to the topic on the timeline. Section 6 briefly summarizes new therapies of neurodegenerative illnesses and, finally, Section 7 provides a summary and conclusions and indicates further trends in research on neurodegenerative disorders.

One of the main goals of this review is to collect concise knowledge of the impact of vanadium on neurodegeneration in humans. It was important to provide objective information about a possible association between vanadium exposure and neurodegenerative disorders in human subjects. In other words, we tried to collect evidence on neurodegenerative alterations in relation to vanadium pollution in humans. Hence, a reliable analysis of the literature data has been carried out and selected literature findings from studies of the neurotoxic effects of this element in man have been overviewed and illustrated in an accessible form to anyone interested in neurodegenerative disorders and vanadium in general. Some results on the neurotoxic effects of this metal in animals have been presented, as well, to offer the reader a deeper insight into studies related to vanadium and neurotoxicity. Additionally, a brief historical framework has been provided to draw the reader’s attention to the research on vanadium in the context of neurodegenerative processes in terms of time.

## 2. Methodology—Literature Search Strategy

### 2.1. Databases: General Outline

The literature search in English-language databases (i.e., PubMed, Scopus, and Web of Science) was conducted from November 2022 to January 2023 to collect relevant data. Only research articles written in English were reviewed and only abstracts in English from papers published in other languages were included. In the case of articles with an unavailable full text, correspondence contact was made, i.e., an email was sent to the corresponding author with a request for sending the full paper. In the absence of a reply, the information provided in the abstracts was included in the current report. To ensure comprehensive search of the literature, the reference lists of selected articles collected from the above-mentioned databases were manually reviewed to identify additional records (i.e., full-text papers or abstracts) that were potentially relevant to the topic.

### 2.2. Query Terms Used for the Literature Search on Neurotoxic Effects of Vanadium in Humans

The search was focused on the ‘Title’ and ‘Abstract’, and such keywords as ‘vanadium or vanadate and Alzheimer disease’, ‘vanadium or vanadate and neurodegeneration’, ‘vanadium or vanadate and dementia’, and ‘vanadium or vanadate and neurodegenerative diseases’ were used to obtain the records on the levels of vanadium in biological specimens, i.e., in the brain, cerebrospinal fluid (CSF), blood, urine, and hair of patients with neurodegenerative illness and on the content of this metal in the whole blood, serum, urine, and CSF of humans exposed to vanadium occupationally, subjects with acute vanadium poisoning and the elderly with neurological side effects and neurobehavioral changes. Additionally, such key terms as ‘vanadium’, ‘particulate matter’, ‘Parkinson disease’, ‘Alzheimer disease’, ‘Amyotrophic lateral sclerosis’, ‘dementia’, ‘neurodegeneration’, ‘neurotoxicity’, and ‘humans’ in various combinations were employed to obtain the data on the neurotoxic effects of vanadium-associated PM_2.5_/PM_10_ exposure in man.

### 2.3. Query Terms Used for the Literature Search on Neurotoxic Effects of Vanadium after Inhalation in Animals

The search terms, focused on the ‘Title’ and ‘Abstract’, such as ‘vanadium’, ‘vanadate’, ‘pollution’, ‘exposure’, ‘accumulation’, ‘neurotoxicity’, ‘animals’, ‘inhalation studies’, ‘brain’, ‘mice’, ‘rat’, ‘animal model’, ‘environmental pollution’, ‘neurodegeneration’, ‘neuroinflammation’, ‘olfactory dysfunction’ and those linked with ‘AND’, were used in the search strategy to obtain the records limited to neurotoxic effects of vanadium after inhalation in animals.

### 2.4. Search Results and Literature Review Flowchart on Neurotoxic Effects of Vanadium in Humans

**Figure 2:**
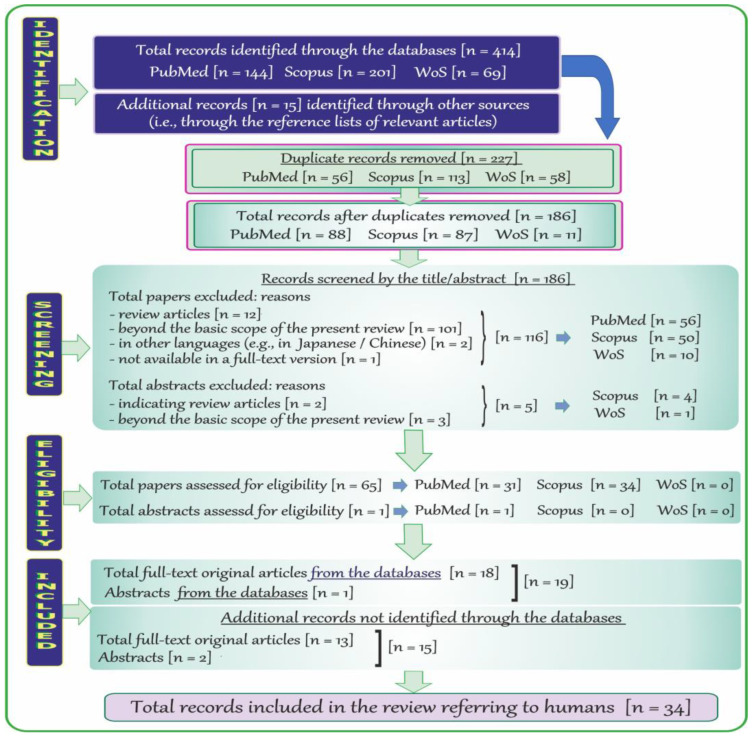
Flow chart of the systematic literature review on neurotoxic effects of vanadium in humans.

The adopted strategy of searching by using specific keywords allowed the detection of records in PubMed (NCBI), Scopus, and WoS relevant to the topic of neurotoxic effects of vanadium in humans. The flow chart provided below (Figure 2) shows the process employed to identify records on neurotoxic effects of vanadium in humans.

A total of 414 records published in English and other languages were identified through the databases listed above; i.e., 144 through PubMed, 201 through Scopus, and 69 through WoS. After removal of 227 duplicate items (PubMed: 56, Scopus: 113, and WoS: 58), the remaining records (n = 186) were initially screened by the titles and abstracts. Afterwards, 121 records published in, e.g., Japanese or Chinese and papers unavailable in a full-text version, as well as review articles and those that did not address the topic were excluded (PubMed: 56, Scopus: 50, and WoS: 10). Moreover, a total of 5 abstracts (Scopus: 4 and WoS: 1) that pointed to review papers, and were far beyond the scope of the present report, were excluded as well. Next, a total of 65 potentially relevant full-text articles (PubMed: 31 and Scopus: 34) and 1 abstract (PubMed) were further examined. Finally, a total of 18 full-text original papers and 1 abstract (n = 19) were included in the current review. Moreover, 15 additional records (i.e., 13 full-text original articles and 2 abstracts) relevant to the topic were included by a manual review of bibliographies.

### 2.5. Search Results and Literature Review Flowchart on Neurotoxic Effects of Vanadium after Inhalation in Animals

**Figure 3:**
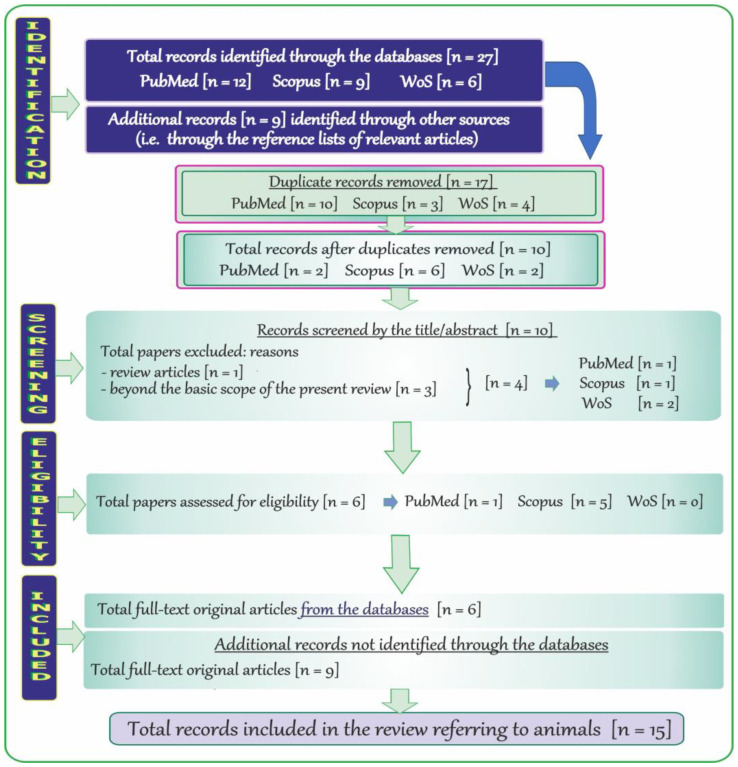
Flow chart of the systematic literature review on neurotoxic effects of vanadium after inhalation in animals.

The adopted strategy of searching by using specific key terms also allowed detection of records in PubMed (NCBI), Scopus, and WoS relevant to the topic of neurotoxic effects of vanadium after inhalation in animals. The flow chart (Figure 3) shows the steps of the methodology described in this subsection.

At the beginning, a total of 27 records published in English were revealed through the databases, i.e., 12 through PubMed, 9 through Scopus, and 6 through WoS. Next, a total of 17 duplicate records (PubMed: 10, Scopus: 3, and WoS: 4) were removed and the remaining items (n = 10; PubMed: 2, Scopus: 6, and WoS: 2) were initially screened by the titles and abstracts. Afterwards, records that were far beyond the topic addressed in the present review were excluded (n = 4; PubMed: 1, Scopus: 1, and WoS: 2), and six potentially relevant items (i.e., only full-text original articles) were further examined. Finally, a total of six full-text original papers in English and nine additional records (i.e., the relevant full-text original articles) were included (n = 15).

## 3. Neurodegenerative Disorders—A Brief Outline with a Polish Accent

Neurodegenerative disorders are currently incurable diseases of the nervous system, which are a constantly growing social concern. They are progressive and lead to gradual degeneration and/or death of nerve cells, which in turn results in cognitive deterioration or impaired motor functions. Due to the lack of satisfactory methods of treatment, neurodegenerative diseases have been arousing interest of many research centers. New therapies that would ensure better treatment results and contribute to a significant slowdown in the progression of the disease are constantly being sought.

### 3.1. Alzheimer Disease (AD)

Alzheimer’s disease (AD) is the most common form of dementia, accounting for 50 to 75% of cases. In Poland, about 500 thousand people suffer from various dementia syndromes, including over half (about 300 thousand) that have been diagnosed with AD. In 30 years this number may triple. The incidence of AD in the age group 65–69 years is 2–3/1000 people/year, but in the range of 85–89 years it is already 37–40/1000 people/year [1].

The etiology of AD is multifactorial and still not fully understood. Only a few forms have a genetic basis (the polymorphism of the gene encoding apolipoprotein E—APOE or mutations in the amyloid precursor protein or presenilin 1 and 2 genes). Most cases are probably related to the deposition of pathological proteins: β-amyloid protein and hyperphosphorylated tau protein. In addition, vascular changes often coexist, which directly damage neurons and stimulate Alzheimer’s degeneration processes [2]. New research draws attention to other mechanisms. Inflammatory processes caused by active microglia, damage to cholinergic neurons, oxidative stress (OS) or glucose metabolism disorders observed in the course of dementia require treating as a systemic disease and paying more attention to the correlation between the brain and other organs [3,4].

So far, approved drugs, including cholinesterase inhibitors, N-methyl-D-aspartate (NMDA) receptor antagonist or their combination, usually provide temporary and incomplete symptomatic relief that may be accompanied by serious side effects. Only in 2021 did a new class of substances join, monoclonal antibodies (aducanumab and two years later lecanumab). Both substances have been approved by the Food and Drug Administration (FDA). Due to controversies regarding their effectiveness and side effects, the European Medicines Agency (EMA) has not yet approved these drugs. However, these substances also only slow down the development of the disease. In addition, their effectiveness is greatest when they are included at the beginning of the disease. Until recently, amyloid and tau protein were the main targets for most drugs in development for AD. It is now believed that it is unlikely that antibodies against amyloid alone will be sufficient to halt or reverse the course of the disease [2].

### 3.2. Parkinson Disease (PD)

Parkinson’s disease (PD) is the second most common neurodegenerative illness. In Poland, about 80,000 people suffer from PD at various stages of advancement. It is estimated that among people over 65 years of age, 1 person in 100 suffers from the disease, while in the population over 85 years of age, 5 out of 100 [5].

Most patients are treated with orally administered levodopa. In addition, carbidopa preparations, dopamine agonists, catechol-O-methyltransferase inhibitors (COMT), monoamine oxidase B inhibitors (MAO-B), anticholinergic drugs and amantadine are used. Although these drugs can bring significant relief in some motor symptoms of PD, additional therapies were needed to solve other serious obstacles regarding quality of life as PD progresses. In terms of new strategies that would allow for better treatment outcomes and significantly slow down the progression of the disease, there are various approaches and therapies, such as gene therapy, cell therapy or recombinant protein therapy. However, most of them are still in the clinical trial phase and are not yet available to patients [6].

### 3.3. Amyotrophic Lateral Sclerosis (ALS)

Amyotrophic lateral sclerosis (ALS) is a rare disease, but it is the most common motor neuron disease among adults [7]. As a result of neurodegenerative processes, there is damage to the corticospinal and corticobulbar tracts, as well as to the motor nuclei of the cranial nerves and the anterior horns of the spinal cord.

The incidence of ALS is small with 1–2 cases per 100,000 people. The number of new cases per year is 4–5 per 100,000 people. In the Polish population, the number of patients can therefore be estimated at around two–three thousand [8].

There are currently two registered drugs (by FDA) for ALS, i.e., riluzole and edaravone. Riluzole slightly prolongs survival time, while edaravone slows down the progression of the disease to a small extent. However, owing to a better understanding of the molecular mechanisms of the disease in recent years, new potential targets for new drugs have been discovered. Details in this field are provided in another section of the present review.

## 4. Vanadium—Selected Issues in a Nutshell

### 4.1. Background

Vanadium (symbol: V) is part of Group 5 (5B) of the periodic table. It is a transition metal existing in a wide range of valence states, but the most common are +2, +3, +4, and +5. As a vanadate anion, i.e., VO_3_− (pentavalent form), it predominates extracellularly and as a vanadyl cation, i.e., VO^2+^ (tetravalent form), it occurs mainly intracellularly [9,10].

Vanadium is widely dispersed in the environment. It occurs in the air, soil, and water in variable concentrations and can be found naturally in about 65 different minerals [11]. It plays a large role in the industrial sector and exhibits multidirectional biological effects. This metal is also essential to some species in trace amounts, but can be toxic in excess. Moreover, in some conditions, vanadium can exert antioxidant properties, as evidenced by animal studies [12,13,14,15]. More details on the issues mentioned above have previously been provided in two review papers [16,17].

**Figure 4:**
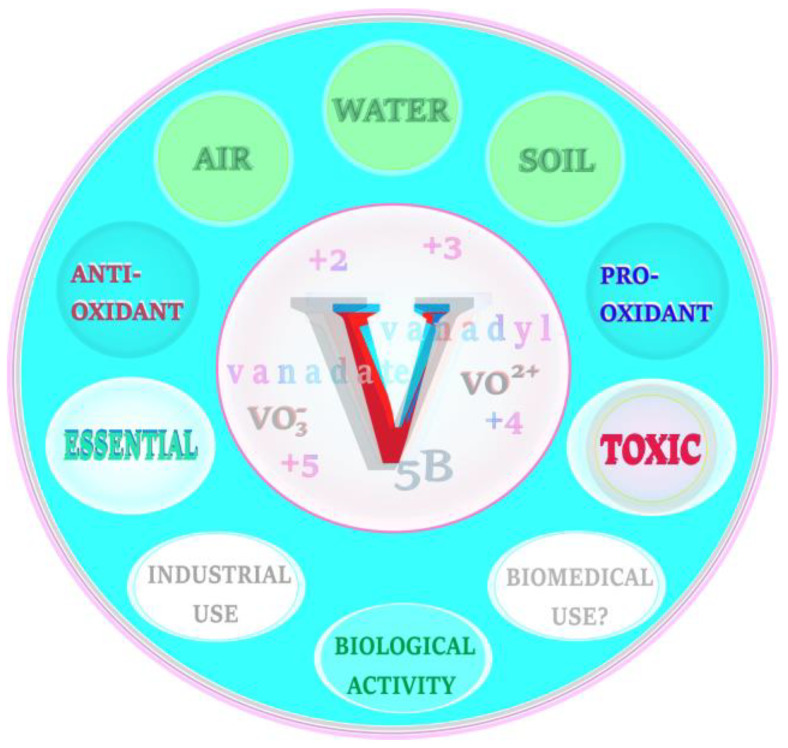
Summary of basic information about vanadium (V). VO_3_−: vanadate anion, VO^2+^: vanadyl cation.

It should be emphasized that vanadium is at the forefront among the different elements studied for their potential therapeutic use, due to its anti-viral, anti-bacterial, anti-parasitic, anti-fungal, anti-allergic, anti-cancer, anti-diabetic, anti-hypercholesterolemic, anti-inflammatory, anti-ulcer, cardio-protective, nephro-protective, and neuro-protective activity [18]. On the other hand, it can accumulate and act as a strong pro-oxidant [19], which limits the use of this metal in the treatment of some modern-age diseases in humans. Therefore, minimization of the adverse effects and pro-oxidative activity of vanadium may contribute to the development of effective therapeutic approaches in which this element could be used. It should also be highlighted that OS, i.e., a deleterious process generated by vanadium, has been reported to be implicated in neurogenerative mechanisms in the pathogenesis of some neurodegenerative disorders [20] that have been described in one of the previous sections of the current report. A brief summary of the background data on vanadium are graphically presented in Figure 4.

### 4.2. The Impact of Certain Factors on the Concentration of Vanadium in Tissues and Body Fluids

**Figure 5:**
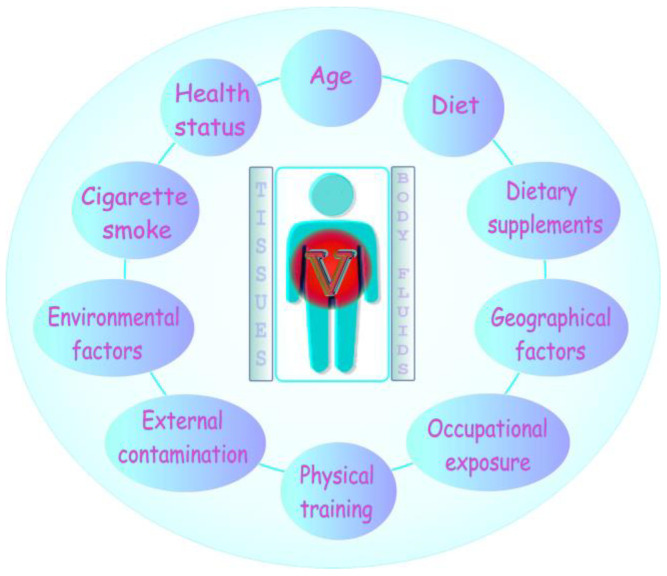
Factors influencing the content of vanadium in tissues and body fluids. Elaborated on the basis of available literature data cited in Section 4.2. V: vanadium.

The level of vanadium in body fluids and tissues is influenced by several factors, such as age, diet, health status, or occupational exposure [11,21,22]. As for the latter, elevated concentrations of this metal with a well-known industrial value [23] have repeatedly been noted in body fluids of vanadium-exposed workers [24]. The environmental vanadium sources and geographical location are important as well [25,26,27], and more details in this field are provided in further section of the present review. External contamination can also play a pivotal role. For example, Minoia et al. [28] observed the release of vanadium, commonly used for steel production [23], from stainless steel needles and syringes during blood collection. Moreover, the presence of vanadium in certain biomedical materials (e.g., in implants), reviewed previously [16], from which vanadium can be released into surrounding tissues may be another factor affecting the level of this metal in the body. Recently, Łanocha-Arendarczyk and co-workers [29] demonstrated a higher concentration of vanadium in spongy bone of the femoral head of patients with osteoarthritis inhabiting urban areas of Western Pomeranian (Poland), who had prosthetic implants and were occupationally exposed to various chemical substances, including heavy metals. Patients with implants also had a higher concentration of vanadium in cartilage [29]. As for the health status, Sampath et al. [30] have recently supported the hypothesis that an imbalance of vanadium (the concentration of which was significantly reduced in the serum following treatment) is a factor in the etiology of one of mental illnesses, i.e., bipolar disorder. Furthermore, dietary supplements frequently used by athletes and body builders to improve performance [31], in which vanadium may be present in the form of, e.g., vanadyl sulfate [11], are equally important. As reported, the prolonged consumption of such supplements, with daily doses up to 18 mg V/day/person, may pose a risk of adverse health effects [31]. Additionally, smoking should be taken into account as one of the determinants of the concentration of vanadium in the organism [32], as vanadium is contained in cigarettes at the mean level of 1.83 µg/g and approximately 30% of this metal is released in the smoke [33]. Therefore, both active and passive smokers may be exposed to higher levels of this element. However, the analysis of the available literature does not reveal any data on a rise in the concentration of vanadium in body fluids resulting from smoking. As reported in a study carried out by Toro-Román and co-workers [34], physical training is another factor that can influence the level of vanadium in the organism. The authors demonstrated that regular physical training, especially aerobic sport modalities, causes an increase in the concentration of this metal in the serum. All the above-mentioned factors are graphically summarized in Figure 5.

### 4.3. Environmental Vanadium Exposure in Humans—Adverse Health Outcomes Excluding Neurotoxic Effects

**Figure 6:**
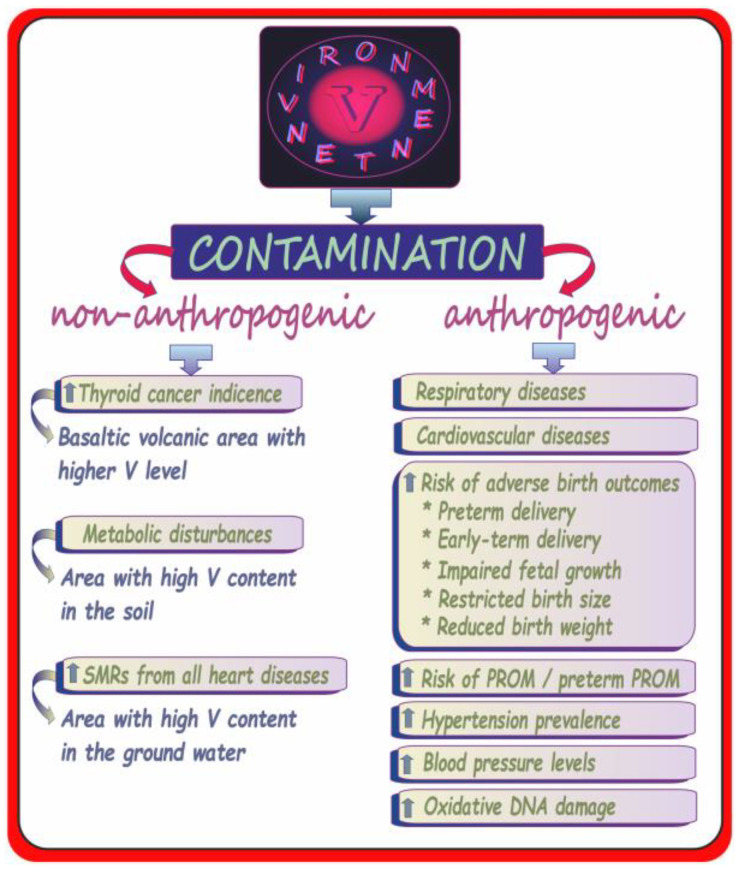
Summary of adverse health outcomes of environmental vanadium exposure in humans excluding neurotoxic effects. Elaborated on the basis of available literature data [25,26,27,35,36,37,38,39,40,41,42,43,44,45]. SMRs: standardized mortality rates, PROM: premature rupture of membranes, V: vanadium. ↑: increase.

The present section is focused on the environmental exposure of humans to vanadium, and offers the reader a concise summary of the potential harmful effects related to both non-anthropogenic and anthropogenic contamination of the environment with this metal (Figure 6). Simultaneously, it refers to the further parts of the current review, in which neurotoxic effects resulting from, e.g., the environmental exposure to vanadium in humans and animals are discussed and where selected issues related to vanadium and neurodegenerative disorders described in the previous sections of the current review are illustrated on the timeline for the reader’s convenience.

As presented in Figure 6, not only anthropogenic human activity resulting in the release of vanadium into the environment but also non-anthropogenic vanadium sources may lead to serious health consequences. As far as the non-anthropogenic contamination with vanadium is concerned, Malandrino et al. [27] determined the concentrations of certain metals (also vanadium) in the tap water and lichens of the Mt. Etna volcanic area (Catania province), where an elevated thyroid cancer incidence has been reported to characterize the environmental pollution of that region, examined the urine of residents inhabiting the area to evaluate the level of biocontamination. The analyses demonstrated an eight-fold higher urinary vanadium concentration in the residents from the volcanic part of the country (0.16 µg/g creatinine) than in those living in the control area: 0.02 µg/g creatinine (i.e., in Palermo and Messina provinces). In turn, the concentration of vanadium in the tap water (19.9 µg/L) was almost 40 times higher than that found in the water from the control region (0.5 µg/L) [27] and often exceeded the maximum admissible concentration [42]. A significant correlation between the urinary vanadium concentration and the water vanadium level was also observed. These data suggest a possible relationship between the pollutants, including vanadium present in the volcanic regions and the increase in the thyroid cancer risk. A higher level of vanadium was also noted in the urine and some internal organs, e.g., kidneys (1.7-fold) and lungs (1.9-fold), in villagers inhabiting northeastern Thailand (where some metabolic problems such as, e.g., distal renal tubular acidosis occur), compared to residents in the central area. Additionally, examinations of the level of vanadium in the soil showed that the surface soil in the northeastern areas exhibited a three-fold higher vanadium concentration than the central part of the country [44]. As suggested by the authors, the high vanadium intake following the prolonged consumption of natural agricultural products and the iron deficiency noted in northeastern Thailand may have led to the elevated tissue and urine vanadium contents. At this point, the findings of the interaction of dietary iron levels on the toxicity of vanadium in chicks conducted by Blalock and Hill [46] are worth mentioning. The authors reported a rise in the degree of toxicity in iron-deficient animals accompanied by an increase in vanadium. More precisely, they showed that the iron-deficient animals retained more vanadium in the blood and liver, but the absorption of this metal was not influenced by the concentration of iron in the diet. In the case of villagers from northeastern Thailand, the concentration of vanadium in the liver was about 1.9-fold higher than in dwellers from the central part of the country [44]. Based on these data, it may be supposed that the accumulation of vanadium in tissues accompanied by iron deficit may have led to the development of renal acidosis in the villagers inhabiting northeastern Thailand. In turn, the work of Seko et al. [43] revealed the highest standardized mortality rates (SMRs) from all heart diseases in one of the eight secondary medical districts in Yamanashi prefecture (i.e., in Fuji-hokuroku, a high-vanadium area) in Japan, where the groundwater used as a source of tap water by habitants is relatively rich in vanadium (its concentration is about 50 µg/L around the mountain). These data allow suggesting an adverse effect of the vanadium-rich groundwater from Mt. Fuji on the health of habitants living around the mountain in that region.

As for the relationship between adverse health outcomes and vanadium contained in particulate matter with a diameter of 2.5 (PM_2.5_), Cakmak et al. [37] reported an association between vanadium in PM_2.5_ and an increased risk of respiratory and cardiovascular diseases. Dai et al. [47], who analyzed the differential effects of PM_2.5_ species and sources on blood markers of inflammation and endothelial dysfunction in a large longitudinal cohort, found that among the species examined (K, S, Se, Al, Si, Fe, Ni, V, Cu, Zn, and Na) vanadium was associated with increased levels of both intercellular adhesion molecule-1 (ICAM-1) and vascular cell adhesion molecule-1 (VCAM-1). Additionally, they noted an association between oil combustion rich in vanadium and ICAM-1 and VCAM-1. Thus, these findings clearly suggest that particles containing vanadium derived from oil combustion may be related to endothelial dysfunction. Moreover, they are important in the context of the studies conducted by Blankenberg et al. [48], who showed that higher levels of ICAM-1 and VCAM-1 are related to future death from cardiovascular causes, and the studies conducted by Pradhan et al. [49], who noted that elevated levels of ICAM-1 are independently associated with the development of accelerated atherosclerosis among otherwise healthy men, even in the absence of acute coronary occlusion. Another study conducted by the Columbia Center for Children’s Environmental Health provided evidence that the exposure to PM_2.5_ associated vanadium and elemental carbon from heating oil and/or traffic at the levels characteristic of urban areas may be linked to asthma morbidity in very young urban children, i.e., at the age of two [41]. Another epidemiological study carried out in the United States, in which the relative risk of hospitalization due to cardiovascular and respiratory diseases in patients at the age of 65 or older was estimated, provided evidence that communities with a higher vanadium content in PM_2.5_ have a higher risk of hospitalizations associated with PM_2.5_ exposure, and the relationship between PM and health varies seasonally and regionally, likewise with the PM_2.5_ chemical composition [36]. Moreover, Chen et al. [50], who examined the association between long-term exposure to PM_2.5_ elemental components and mortality in a large pooled European cohort, found that the long-term exposure, especially to vanadium in PM_2.5_, was associated with an increased mortality risk. Wang et al. [51] also observed an increased risk of mortality upon long-term exposure to PM_2.5_ with higher vanadium content among older adults in the southeastern US. In turn, in a study including 11 European cohorts, Wolf et al. [52] found a positive association between long-term exposure to vanadium-containing PM and the incidence of coronary events. A positive association between long-term exposure to vanadium-containing PM_2.5_ and the lung cancer incidence, as well as a negative association between vanadium-containing PM_2.5_ and lung function, were reported as well [53,54]. As suggested by Alfaro-Moreno et al. [55], “*changes in the concentration of PM components help explain the changes in biological effects and support the hypothesis that particle composition plays an important role in particle-induced toxicity*”. Studies conducted by these authors aimed to determine the relationships between specific components present in PM_10_ and cell viability, cytokine secretion, DNA damage, and E-selectin expression to test the hypothesis that the composition plays an important role in PM effects. They identified a strong negative association of vanadium with cellular viability and a strong positive association of this metal with cytokine production, i.e., interleukin 6 (IL-6) and tumor necrosis factor (TNF-α) as well as a discrete positive association with the comet longitude. The study conducted by Campen et al. [56], who investigated the effects of inhaled PM-associated transition metals (V and Ni), also supported the hypothesis of the adverse health effects exerted by metals contained in PM air pollution and provided insight into potential interactions regarding the toxicity of PM-bound elements. Interesting findings were obtained by Sørensen et al. [45] who examined the relationship between the personal exposure to some transition metals such as V, Cr, Fe, Ni, Cu, and Pt in PM_2.5_ and oxidative DNA damage in human volunteers (i.e., in 49 students from central Copenhagen). The authors indicated that, among the six examined metals mentioned above, only vanadium and Cr were associated with the 7-hydro-8-oxo-2′-deoxyguanosine (8-oxodG) level in lymphocytes independently of the PM_2.5_ mass. Thus, these results clearly suggest that vanadium and Cr contained in PM_2.5_ can induce oxidative DNA damage. As stressed by the authors, the effect was independent of particle mass and/or other toxic compounds present in the particulate mixture. In turn, Basu et al. [35], who studied the influence of fine particulate matter and its constituents on low birth weight among full-term infants in California, demonstrated that the exposure to specific constituents of PM_2.5_, especially such metals as vanadium, is associated with the largest reductions in birth weight. Moreover, a study conducted by Jin et al. [40] among 7290 pregnant women from China focused on evaluation of the relationship between urinary metal concentrations (including vanadium) and the risk of the premature rupture of membranes (PROM), revealing a positive association of the urinary vanadium level with PROM and preterm PROM. These results suggest that pregnant women who are exposed to higher levels of vanadium (in urban areas of China) have an increased risk of PROM and preterm PROM. Additionally, Jiang et al. [38] conducted a nested case–control study in the province of Hubei (China) to investigate the relationship between maternal urinary vanadium concentrations and the odds of delivering low birthweight (LBW) infants. The study revealed that higher levels of maternal urinary vanadium were associated with increased odds of delivering LBW infants. The authors pointed to the need for further studies on the urinary vanadium concentration during pregnancy to monitor changes in the level of vanadium during pregnancy and to assess the critical exposure-time window for the effect of vanadium exposure in fetal development. Further, the work conducted by Hu et al. [25] to estimate the association of prenatal exposure to vanadium with the risk of adverse birth outcomes in babies born to women in China, demonstrated a relationship between the prenatal exposure to vanadium and the increased risk of preterm delivery and early term delivery. Another study conducted by Hu et al. [26] to assess trimester-specific associations of vanadium exposure with ultrasound measures of fetal growth and birth size in a Chinese longitudinal cohort, indicated differences in the associations between prenatal exposure to vanadium and fetal growth depending on the time of exposure, and suggested that the first, early second, and late third trimester can be critical windows of vanadium exposure for fetal growth. Finally, Jiang et al. [39], who studied the association of vanadium exposure with hypertension prevalence and blood pressure levels in a general Chinese population, found that urinary vanadium concentrations were significantly associated with increased hypertension prevalence and blood pressure levels. In view of these results, the authors point to the need to pay more attention to vanadium, which is well known to enter the environment from various types of natural and anthropogenic sources. As vanadium has a role in contamination-related health risks, the necessity of monitoring areas with elevated vanadium concentrations has been repeatedly emphasized. Some researchers also propose the use of certain organisms as natural bioindicators of environmental vanadium pollution, and potential human health problems associated with vanadium exposure may act as an early warning system for hazards. This issue has been concisely summarized previously [17]. To sum up, the data described above clearly indicate that the geographical location and elevated amounts of vanadium in the environmental media (i.e., the air, water, or soil) may have a serious impact on public health.

## 5. Vanadium Neurotoxicity—Overview of Selected Findings: Humans and Animals

### 5.1. Vanadium in Biological Samples of Humans with AD, PD, and ALS—A Summary of Data on the Timeline

**Figure 7:**
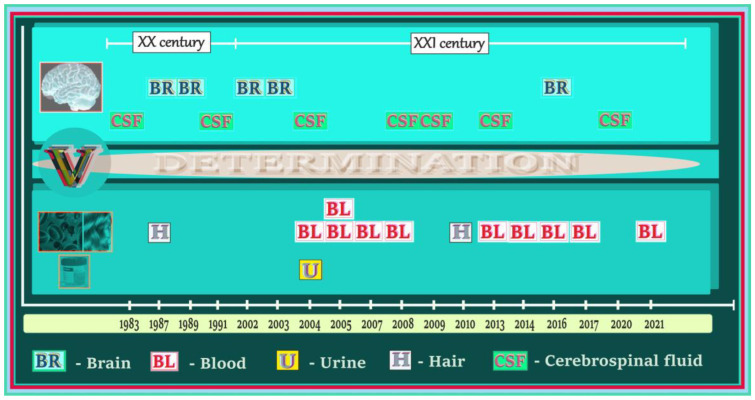
Summary of studies on the levels of vanadium in biological specimens in humans with certain neurodegenerative disorders on the timeline. Elaborated on the basis of data cited in Section 5.2 and Section 5.3.

A summary of studies on the levels of vanadium in biological specimens from humans with such neurodegenerative diseases as AD, PD, and ALS is presented in Figure 7. As illustrated, brain (BR), cerebrospinal fluid (CSF), blood (BL), urine (U), and hair (H) were tested to determine the vanadium content. Among the above-mentioned biological samples, BL was used most often for determination of the concentration of vanadium in patients with neurodegenerative illnesses, BR and CSF were used at a comparable frequency, and U and H were the least often tested in terms of accumulation of vanadium in subjects suffering from neurodegenerative disorders.

The first reference on the detection of vanadium in biological samples (i.e., CSF) obtained from patients with one of the neurodegenerative diseases, i.e., AD, appeared at the end of the 20th century, in 1983. Generally, in the 1980s and 1990s, there were not many investigations based on measurements of vanadium in biological specimens from subjects suffering from neurodegenerative illnesses. More papers on vanadium accumulation in this research area have been published since the beginning of the 21st century, which may reflect the increasing interest in the effects of vanadium on the development of neurodegenerative processes. To date, a total of 21 articles on the level of vanadium in AD, PD, or ALS patients have been issued. Except for five papers published in the 20th century, i.e., in 1983 (CSF), 1987 (BR), 1987 (H), 1989 (BR), and 1991 (CSF), sixteen articles were issued in the 21st century: in 2002 (BR), 2003 (BR), 2004 (CSF/BL/U), 2005 (BL), 2005 (BL), 2007 (BL), 2008 (CSF/BL), 2009 (CSF), 2010 (H), 2013 (CSF/BL), 2014 (BL), 2016 (BR), 2016 (BL), 2017 (BL), 2020 (CSF), and 2021 (BL).

### 5.2. Vanadium Content in Brain and Cerebrospinal Fluid of Patients with AD, PD, and ALS

Summarized values of the concentrations of vanadium in the different parts of the brain and in CSF are listed in Table 1.

As for the level of vanadium in CSF of patients with AD, PD, or ALS, Gerhardsson et al. [58,61] found a significantly lowered concentration of this metal in CSF in subjects with AD, compared with the control. As emphasized by the authors, the observed effect could be related to the binding of vanadium to chelating proteins like Aβ and apolipoprotein E (APOE) in the CSF and brain, thereby detoxifying its pro-oxidative activity. In turn, Hershey et al. [59] did not detect vanadium in CSF of demented Alzheimer patients. In the case of PD, significantly reduced vanadium content was noted by Bocca et al. [67] in CSF of PD subjects, in comparison with the control. However, another study conducted by Shi et al. [66] who determined multiple chemical elements, including vanadium in CSF of subjects suffering from Parkinson disease before and after intracerebral autotransplantation of the adrenal medulla, showed a significantly higher level of vanadium in the 1st, 2nd, 4th, 6th, and 8th week. As concluded by the authors, the observed changes could be induced by the type of operation used. As for ALS, Roos et al. [63] noted a significantly elevated vanadium concentration in CSF of patients with ALS, whereas Patti et al. [65], who compared metal levels in CSF of ALS subtypes (i.e., spinal vs. bulbar clinical onset), did not find any significant alterations in the concentrations of vanadium in CSF between spinal and bulbar onset patients.

As far as the level of vanadium in the brain of AD, PD, or ALS patients is concerned, studies conducted by Szabo and co-workers [57] to examine the relationship between different metal concentrations, including vanadium in the brain and ventricular fluid (VF) of AD subjects and nondemended elderly controls, did not reveal any significant changes in the content of this metal in the frontal cortex; vanadium was not detected in VF. In turn, Ward and Mason [60] conducted studies to investigate many metals, including vanadium, in the brain tissue, i.e., in the hippocampus and cerebral cortex (CC) of Alzheimer’s disease patients and age-matched controls from Eastern Canada and the United Kingdom. They demonstrated a significantly lowered vanadium concentration in both study areas of the brain of the AD subjects, compared to the control. Given the imbalance of other metals (i.e., Br, Ca, S, Si, Se, and Zn) found by the authors in the brain of the AD individuals, it can be assumed that the changes in the level of vanadium and the above-mentioned elements may have resulted from the altered brain metabolism in the response to pathogenic conditions. Another example was reported by Srivastava and Jain [62], who focused on elemental analysis in two AD brain regions, i.e., the parietal cortex and the cerebellum. In this case, however, the concentrations of vanadium are difficult to comment, as they were presented together with other metals in the graph, whose scale does not allow reading the concentration of this element correctly due to its too low values. Further, other researchers analyzing the regional metal concentration, including vanadium in the control and PD brain, detected vanadium in the caudate nucleus (CN) in only one PD brain but the exact levels of this metal in the CN of the PD and control brains were not provided [68]. Finally, Gellein and co-workers [64] carried out studies on the concentrations of selected metals, including vanadium, in formalin-fixed brain tissue collected in 1979–1983 from eight Guamanian patients with ALS, four subjects with Parkinsonism-dementia complex (PDC) and five with no known neurological disorders. The authors did not find any significant differences in the level of vanadium in the different brain regions between both patients groups and the control individuals. As stressed by the researchers, the limited number of subjects, inter alia, may have been responsible for the absence of statistically significant differences (Table 1).

### 5.3. Vanadium Content in Blood, Urine, and Hair of Patients with AD, PD, and ALS

Summarized values of the concentrations of vanadium in blood (including serum (S), plasma (P), and whole blood (WB)) and in urine (U) and hair (H) are listed in Table 2.

As shown in Table 2, the studies conducted by Bocca et al. [67] did not indicate any significant differences in the level of vanadium in the serum of AD patients, compared to the control. In turn, in the case of WB, the concentration of vanadium in AD subjects was noticeably higher (by 1.4-fold), in comparison with the control, but this increase did not reach a statistically significant level. A clear trend toward an increase in the serum vanadium level in AD patients, compared to healthy control, was also observed by González-Dominguez et al. [69]; in this case, the concentration of vanadium in the serum of AD subjects turned out to be about 1-fold higher than that noted in the control individuals. Moreover, the same researchers showed that the level of vanadium in the serum of subjects with mild cognitive impairment was clearly higher (1.1-fold) than in the control, but slightly lower than that in the AD patients. Based on these findings, it cannot be excluded that the concentration of vanadium in the serum of AD patients may reach a significant level with the progress of the disease. Guan et al. [73] also found a higher, but not statistically significant, level of vanadium in the plasma of the AD group, compared to the control. In turn, a statistically significant increase (by 2-fold) in the concentration of vanadium in the serum of AD patients, compared to healthy subjects, was recorded by Paglia et al. [70]. These authors also indicated that the level of vanadium in the serum was significantly elevated in subjects with mild cognitive impairment. Additionally, they revealed that vanadium had the best diagnostic power in the discrimination between AD and healthy individuals. Lavanya et al. [72] also noted a statistically significant increase (by 1.9-fold) in the concentration of vanadium in the serum of AD patients, compared to the control. However, the concentrations of this metal in the serum reported by these researchers are slightly surprising. To sum up, on the basis of the findings described above, it can be suggested that vanadium may play a role in the pathogenesis of AD disease. However, the studies conducted by Gerhardsson et al. [58], Akanle et al. [71], and Alimonti et al. [74] analyzing the level of vanadium in the plasma, hair, and serum of subjects with AD, respectively, did not reveal any significant alterations in the concentration of this metal.

As for the Parkinson disease, the studies conducted by Bocca et al. [67] indicated a significant increase in the concentrations of vanadium in WB and U in PD patients and a clear trend toward an increase in the level of this metal in the serum of these subjects, compared to the control. The levels of vanadium in the PD cases were about 3-fold, 2-fold, and 1-fold higher, respectively, in comparison with those noted in the control individuals. Moreover, the serum vanadium level was found to be positively correlated with the serum antioxidant status, which turned out to be lower in the PD patients than in the control. Further, a statistically significant increase (1.8-fold) in the level of vanadium in the serum in PD patients, compared to healthy individuals, was found by Forte et al. [76]. However, the authors demonstrated that antioxidant capacity and oxidative damage were reduced and elevated, respectively, in PD subjects. An increased level of vanadium in the serum (1.8-fold) in PD patients, compared to control subjects, and reduced antioxidant capacity and enhanced oxidative status in the serum of these patients, were also reported by Alimonti et al. [74]. Thus, the results described above clearly point to alterations in the level of vanadium in body fluids of PD patients and do not rule out the involvement of this metal, which is able to generate OS in the pathogenesis of Parkinson disease. Simultaneously, these findings indicate that vanadium may be considered as a potential biomarker of this neurodegenerative syndrome and suggest that it can serve as a predictor of PD evolution and treatment of this illness.

As far as ALS is concerned, to date, there are only two papers presenting the levels of vanadium in ALS subjects and healthy controls. For example, Royce–Nagel et al. [77] who determined the level of vanadium in hair, did not find elevated levels of this metal in hair samples from individuals with ALS. Therefore, based on these findings, they concluded that hair vanadium levels cannot serve as a biomarker of primary pathogenic events in ALS. Similarly, no significant changes in the level of vanadium in the serum of ALS subjects, compared to the control, were noted by Roos and co-workers [63]. However, in this case, the authors recorded disturbances in the concentration of this metal in CSF of ALS patients, which has already been described in the previous section of the present report.

### 5.4. Vanadium in Biological Samples of Humans Exposed to Vanadium Occupationally, Subjects with Acute Vanadium Poisoning and the Elderly with Neurological Side Effects/Neurobehavioral Changes—A Summary of Data on the Timeline

**Figure 8:**
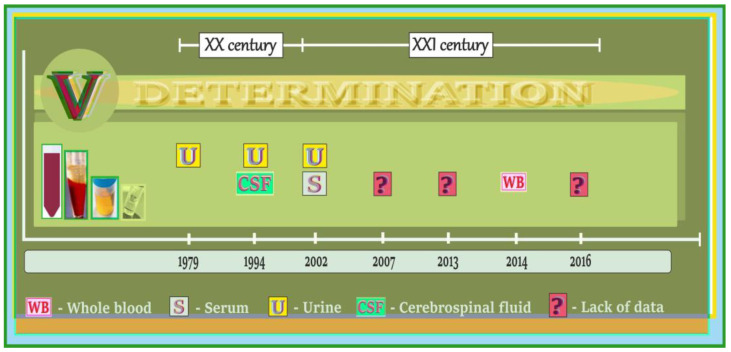
Summary of studies on the levels of vanadium in some biological specimens in humans exposed to vanadium occupationally, subjects with acute vanadium poisoning and the elderly with noted neurological side effects/neurobehavioral alterations on the timeline. Elaborated on the basis of data cited in Section 5.5.

A summary of studies on the levels of vanadium in biological specimens from humans exposed to vanadium occupationally, subjects with acute vanadium poisoning, and the elderly with neurological side effects and certain neurobehavioral alterations is presented in Figure 8. As illustrated, CSF, WB, S, and U were tested to determine the vanadium content.

To our knowledge, the first report on the level of vanadium in a biological sample, i.e., in the urine (U) of patients with some neurological disorders, was published in the late 20th century, i.e., in 1979, and referred to workers occupationally exposed to this metal. Several years later, in 1994, another paper appeared in which the concentration of vanadium was recorded in the CSF and U of female patients with acute vanadium poisoning and certain neurological alterations. In the 21st century, there are not many investigations in this research field. More precisely, only one paper on the levels of vanadium in the S and U of occupationally exposed subjects with neurological changes was published in 2002. In subsequent years, i.e., in 2007, 2013, and 2016, three articles described some neurological side effects in occupationally exposed workers, but no information about the concentration of vanadium in physiological fluids of these persons was provided. Finally, in 2014, one paper was published on the level of vanadium in the WB of elderly subjects with some neurological alterations. More details on the type of neurological side effects and neurobehavioral changes along with summarized values of the concentration of vanadium in some biological samples from the subjects mentioned above are provided below.

### 5.5. Neurological Side Effects/Neurobehavioral Changes and Vanadium Content in Body Fluids of Humans Exposed to Vanadium Occupationally, Subjects with Acute Vanadium Poisoning and the Elderly

The present section provides a brief outline of the neurological side effects/neurobehavioral alterations of vanadium in humans. Details on this topic are concisely summarized in Table 3 and described below.

As shown in Table 3, one of the studies was conducted by Barth et al. [78] who used a modified Wisconsin Card Sorting Test, block design test, visual recognition test, simple reaction time, choice reaction, digit symbol substitution, and digit span to examine the effects of vanadium on attention, visuospatial and visuomotor functioning, reaction time, short-term memory, and prefrontal functioning in occupationally exposed subjects. The results of this study showed that vanadium, at the concentration of 14.2 µg/L in the urine, reduced neurobehavioral abilities, particularly visuospatial abilities and attention. A significant correlation between urinary and serum vanadium levels and cognitive deficit was also noted [78]. The concentrations of vanadium in the serum and urine of the occupationally vanadium-exposed people participating in these studies were about 9.4-fold and 36-fold higher, respectively, compared to those noted in the nonexposed subjects. Moreover, the serum and urinary vanadium levels in the same exposed workers were about 50-fold and 48-fold higher, compared to the most probable mean baseline serum (~0.15 µg/L) [84] and urinary (0.2–0.4 ng/mL) [21] vanadium concentrations, respectively, reported for humans not exposed to this metal. Another example is the results reported by Li et al. [80], who tested the hypothesis that occupational exposure to vanadium in a low-dose long-term exposure condition may lead to an early onset of neurobehavioral changes in vanadium-exposed Chinese workers. The neurobehavioral tests used by the authors, such as simple reaction time, digit symbol, Santa Ana dexterity, digital span, Benton visual retention, and pursuit aiming, included in the World Health Organization recommended Neurobehavioral Core Test Battery (WHO-NCTB), which is designed to identify adverse behavioral effects of chemical intoxication in the human population [85], pointed to poorer performance among exposed workers, compared to unexposed control subjects. The exposed workers exhibited increased anger-hostility, depression-dejection, and fatigue-interia and decreased vigor-activity. Longer mean reaction times and more counting errors were also observed in the exposed group. Moreover, vanadium exposure has been found to be associated with decreased coordination, auditory memory, and perception/motion speed. Unfortunately, no information about the levels of vanadium in the blood or urine of workers participating in these studies was provided. Further, with the use of the Santa Ana test, Benton visual retention test, and pursuit aiming II test, Zhou and co-workers [81] also investigated the effect of vanadium on neurobehavioral functions in workers exposed to this metal. The study revealed that the vanadium-exposed subjects showed poorer performance in the above-mentioned tests. The results obtained allow a conclusion that workers exposed to vanadium may show mood disorders, decreased vision-memory, and lower motor-speed and accuracy. Zhu et al. [82], who examined the effect of vanadium exposure on neurobehavioral function in workers with the use of WHO-NCTB tests as well (i.e., digit span, digit symbol, Santa Ana, Benton visual retention, and pursuit aiming II), found that the group exposed to this metal had lower test scores than the control. Their results indicate that the exposure to vanadium can manifest itself in decreased hearing/visual memory, movement, velocity, accuracy, and coordination. However, similarly in the studies conducted by Li et al. [80], no information about the levels of vanadium in the blood or urine of workers was provided. Compiled, the findings described by the above-mentioned authors clearly indicate that occupational exposure to vanadium can have adverse effects on neurobehavioral function.

In turn, studies conducted by Baierle et al. [22] to examine whether age-related cognitive deficit is associated with oxidative damage (especially with delta-aminolevulinate dehydratase inhibition, ALA-D) and to verify the influence of some metals, including vanadium on ALA-D activity and cognitive performance, showed that an increased level of vanadium was associated with reduced cognitive ability in Mini-Mental State Examination (MMSE) in an elderly group. Moreover, vanadium was noted to be negatively associated with ALA-D reactivation. Thus, the results of this study indicated that vanadium may contribute to a cognitive decline. It should also be mentioned that the concentration of vanadium in the whole blood of elderly subjects (i.e., 24.44 µg/L) was above that noted in serum of vanadium non-exposed humans (~0.15 µg/L; 0.016–0.939 µg/L) [84]. Another study conducted by Usutani et al. [79] on special physical examination of vanadium handling workers demonstrated tremor of fingers in the occupationally subjects with elevated levels of vanadium. More precisely, the concentration of this metal in the urine of these workers reached the value of 15.5 µg/L (3.0–35.2 µg/L). Finally, right-sided brachiofacial paresis, right hemihypesthesia, right sensorimotor hemiparesis, and mixed amnestic and sensorimotor aphasia were reported in a 22-year old woman who attempted suicide by oral ingestion of ~10–15 g ammonium metavanadate [83]. The concentration of vanadium in the urine of this woman 3 and 9 months after detoxification therapy (i.e., 61.5 µg/L and 12.3 µg/L) was about 205-fold and 41-fold higher, respectively [83], compared to the most probable mean baseline urinary vanadium levels, i.e., 0.2–0.4 ng/mL [21].

### 5.6. Vanadium and Neurotoxic Outcomes in Humans and Animals—A Summary of Data on the Timeline

**Figure 9:**
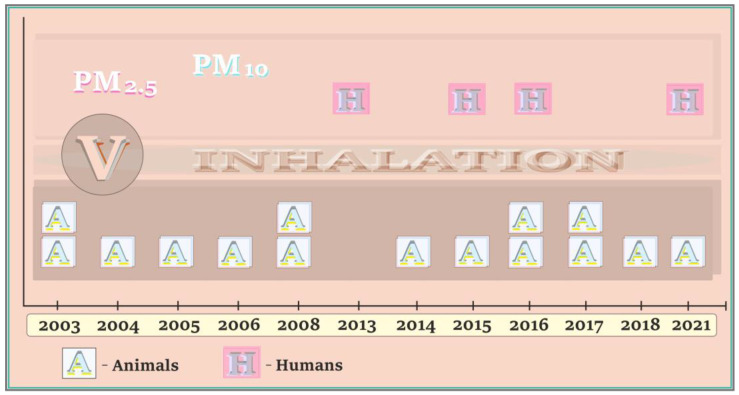
Summary of studies on neurotoxic effects of vanadium in humans and animals after inhalation on the timeline. Elaborated on the basis of data cited in Section 5.7 and Section 5.8.

A summary of studies on the neurotoxic effects of vanadium in humans exposed to vanadium-bound PM_2.5_/PM_10_, and in animals after inhalation, is illustrated in Figure 9. As shown, there are only a few literature reports describing the effects of neurotoxicity related to the exposure of humans to vanadium-bound PM_2.5_/PM_10_. They were published in 2013, 2015, and 2016 by Calderón–Garcidueña’s research group [86,87,88] and in 2021 by Yu and co-workers [89]. More articles in the analyzed research field, i.e., 15 in total, refer to animals exposed to vanadium through inhalation. Three of these papers (published in 2003, 2008, and 2017) refer to animals naturally exposed to urban air and the other twelve reports (published in 2003, 2004, 2005, 2006, 2008, 2014, 2015, 2016, 2017, 2018, and 2021) provide results of the studies on laboratory animals (rats and mice). More details on the neurotoxic effects of vanadium in humans exposed to PM_2.5_/PM_10_ rich in this metal, as well as in animals, are concisely summarized in further sections of this review.

### 5.7. Neurotoxic Effects of Environmental Vanadium Exposure in Humans—A Summarizing Note

The potential negative impact of specific air pollution components on neurodegenerative disorders, including AD, PD, and ALS, has received more attention. Some reports indicate that certain environmental factors, including metals, are considered to increase the risk of these illnesses [90,91,92].

Vanadium, the environmental exposure to which occurs, inter alia, via inhalation [93,94], is a component of fine-particulate air pollution [37] posing a serious public health concern. This metal is present in both the PM_2.5_ and PM_10_ fraction and is mainly associated with mixed industrial/fuel oil combustion [95]. It should be emphasized that the combustion of heavy fuels, especially in oil-fired power plants, refineries, and industrial boilers, and coal are the major anthropogenic sources of atmospheric emission of vanadium [96,97], which is three times higher compared to vanadium releases from natural sources [98]. The findings obtained by Dye et al. [99] in cultured airway epithelial cells exposed to residual oil fly ash (ROFA), a component of ambient PM, revealed that vanadium was largely responsible for ROFA toxicity and that this metal induced its cellular effects, at least in part, through the generation of OS implicated in the progression of neurodegenerative diseases, as mentioned previously.

Since vanadium is a well-known environmental and occupational pollutant, the recognition of possible side effects resulting from the exposure to this metal in the context of neurodegenerative syndromes is crucial. Therefore, a possible association of vanadium with AD, PD, and ALS has been discussed in the present section, as it may provide valuable information on the potential pathophysiology of neurodegenerative illnesses. In other words, the main aim of this part of the review, which supplements the findings presented in the previous part of the current report on the association between vanadium in PM and an increased risk of adverse health outcomes excluding neurodegenerative changes in humans, is to summarize the data on a possible role of this metal in unbeneficial effects on the central nervous system (CNS) in humans. We believe that the findings presented in this chapter, that were gathered based on the literature search, will be useful to those interested in the effects of vanadium on neurodegeneration and increase the awareness of the environmental impact of this element on health.

**Figure 10:**
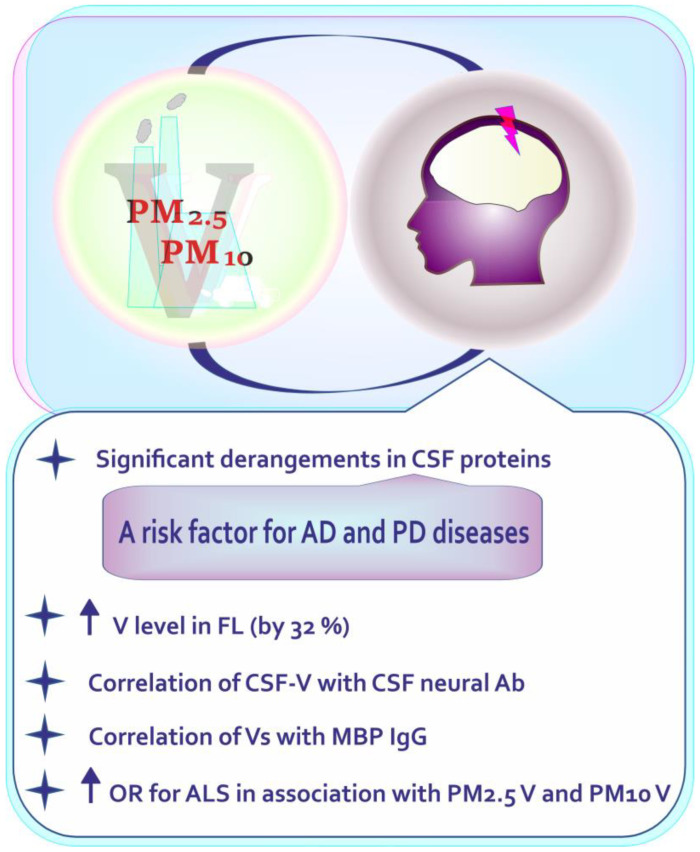
Summary of data on the neurotoxic effects of vanadium-associated PM_2.5_/PM_10_ exposure in humans. Elaborated on the basis of available literature data [86,87,88,89]. PM_2.5_: particles with aerodynamic diameters less than 2.5 µm, PM_10_: particles with aerodynamic diameters less than 10 µm, PM_2.5_V: PM_2.5_ vanadium fraction, PM_10_V: PM_10_ vanadium fraction, CSF: cerebrospinal fluid, CSF-V: CSF-V concentration, V_S_: vanadium in the serum, MBP: myelin basic protein, IgG: immunoglobulin G, FL: frontal lobe, Ab: antibody, AD: Alzheimer disease, PD: Parkinson disease, OR: odd ratio; ALS: amyotrophic lateral sclerosis, V: vanadium. ↑: increase.

Although the number of studies on environmental vanadium-bound PM pollution and its possible deleterious neurocognitive consequences in humans is limited (Figure 9), they deserve our comments. Figure 10 summarizes data on neurotoxic outcomes of vanadium-associated PM_2.5_/PM_10_ exposure in humans.

For example, Calderón –Garcidueñas et al. [86] examined the content of metals (including vanadium) resulting from anthropogenic activity in the frontal cortex of subjects residing in high and low pollution areas, i.e., in Mexico City (one of the world’s most polluted city in the world) and in Tlaxcala and Veracruz (two control cities). The researchers found a higher (by 32%) concentration of vanadium in the frontal cortex in Mexico City subjects chronically exposed to environmental PM_2.5_ containing industrial metals than in the subjects from control localities. The Mexico City residents also had higher levels of inflammatory mediators, i.e., cyclooxygenase-2 (COX2) mRNA and interleukin 1β (IL-1β) in the frontal cortex and COX2 in the olfactory bulb. Based on these findings, it can be suggested that humans exposed to a polluted urban environment rich in industrial PM_2.5_-associated metals, including vanadium, which in some conditions may act as a pro-inflammatory agent [100] and induce the expression of COX2 mRNA [101], face a higher risk of neuroinflammation and detrimental effects on CNS. Another study conducted by Calderón–Garcidueña’s research group [87] was focused on the examination of serum and CSF antibodies to neural and tight junction proteins and environmental pollutants in 139 children aged ~12 year living in Mexico City. They showed that the CSF-vanadium level in the Mexico City children significantly correlated with the CSF neural antibodies (S100 IgA/IgG). Moreover, the authors observed a correlation between the concentration of V in the serum (Vs) and myelin basic protein (MBP) IgG in control children with an elevated serum vanadium level which, as they stressed, could be related to emissions from the Tula refinery [87]. At this point, the results of studies conducted by Todorich et al. [102] and Soazo et al. [103] in a rodent model are worth mentioning. The researchers found that exposure to vanadium during early brain development produces hypomyelination [102], and postnatal vanadium intoxication leads to CNS myelin deficiency [103]. Moreover, Azeez and co-workers [104], who examined regional myelin and axon damage and neuroinflammation in adult mouse brains after long-term postnatal vanadium exposure, observed myelin damage which involved the midline corpus callosum and fibers in cortical gray matter, hippocampus, and diencephalon associated with axonal damage. Significant induction of TNF and IL-1β in the brain was noted as well. Similarly, Usende et al. [105] also observed intense destruction of myelin sheaths in vanadium exposed rats. As known, “*myelination is important in establishing connectivity in the growing brain by facilitating rapid and synchronized information transfer across the nervous system, which is essential to higher-order cognitive functions*” [106]. Therefore, due to the results obtained by Calderón–Garcidueñas and co-workers mentioned above and the findings obtained by Todorich et al. [102], Soazo et al. [103], Azeez and et al. [104], and Usende et al. [105], from a rodent model focused on the influence of vanadium on CNS, it should not be excluded that the exposure of industrial PM_2.5_-associated V in humans can increase the risk of neurological/cognitive disorders. The studies carried out by Calderón–Garcidueñas and co-workers [88] also demonstrated significant derangements in CSF proteins, crucial for the development of AD and PD, in highly PM_2.5_-exposed young Mexico City residents, compared to controls. The authors found that the concentration of amyloid-β_1-42_ and brain-derived neurotrophic factor (BDNF) were significantly lowered. BDNF plays an important role in the proper functioning of the nervous system [107]. It is involved in plastic changes related to learning and memory and its reduced level has been reported to be associated with pathological conditions, including AD and PD [108]. At this point, the results obtained by Wang et al. [109], who determined the effects of exercise on the motor coordination in lactational V-exposed rats should be quoted. The researchers recorded impaired motor coordination and reduced plasma and cerebellum BDNF in the vanadium-intoxicated animals. Further, Calderón–Garcidueñas and co-workers [88] also observed an increase in total-α-synuclein (T-α-Syn) in the first childhood years related to cumulated PM_2.5_ and a decrease after the age of 12 years, whereas d-α-synuclein exhibited a trend toward an increase with cumulated PM_2.5_ [88]. Additionally, in Mexico City children, amyloid-β_1-42_ correlated with T-α-Syn, and d-α-synuclein (d-α-Syn) correlated with tumor necrosis factor alpha (TNFα) and such interleukins as IL-6 and IL-10. Taken together, it can be suggested that young subjects living in highly industrialized areas and exposed to higher concentration of PM_2.5_ rich in vanadium may face a higher risk of neurodegenerative syndromes. González–Maciel et al. [110] also identified combustion-derived nanoparticles (CDNPs) in neurons, glia, choroid plexus, and neurovascular units in young Mexico City residents; they were associated with pathology in mitochondria, endoplasmic reticulum (ER), mitochondria-ER contacts, axons, and dendrites. Finally, Yu et al. [89], who examined long-term exposure to ultrafine particles and PM constituents and the risk of ALS, observed increased odds ratios (ORs) for ALS in association with most air pollutants with the strongest associations for PM_2.5_. They also found that, in the case of particle elements, road traffic non-tailpipe emissions of vanadium as well as Cu, Fe, Ni, S, and Si were associated with significantly higher ORs for ALS in both the PM_2.5_ and PM_10_ fraction. In turn, Cole-Hunter et al. [111], who examined the association between long-term exposure to ambient air pollution and PD mortality in seven European cohorts, provided evidence that long-term exposure to PM_2.5_ at levels well below current EU air pollution limit values may contribute to PD mortality. However, no positive association with the vanadium component of PM_2.5_ was detected.

### 5.8. Neurotoxic Effects of Vanadium after Inhalation in Animals—A Summarizing Note

**Figure 11:**
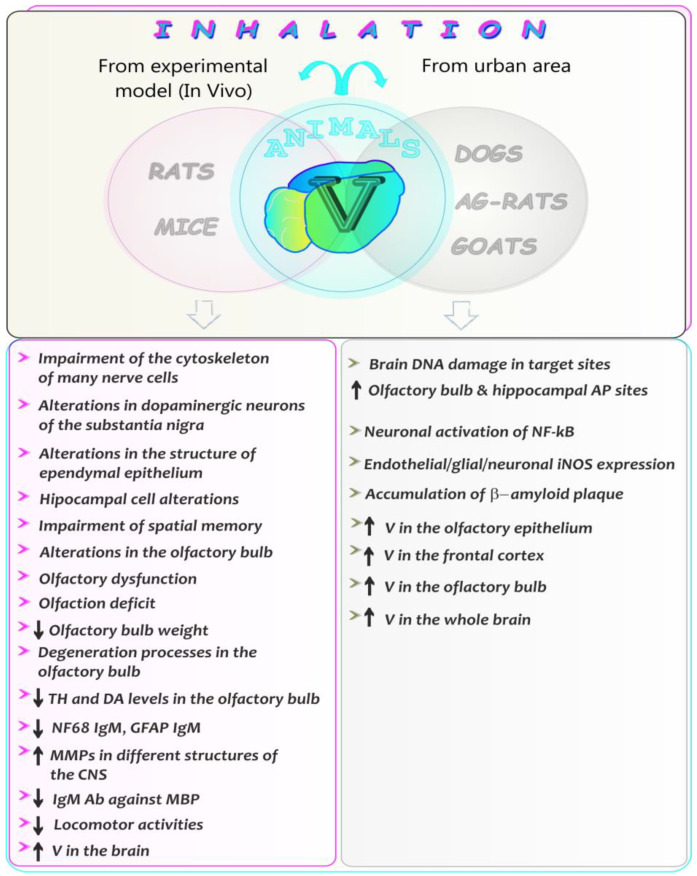
Summary of neurotoxic effects of vanadium after inhalation: studies in laboratory animals and those naturally exposed to urban air. Elaborated on the basis of the available literature data [112,113,114,115,116,117,118,119,120,121,122,123,124,125,126]. Ab: antibody, AG-rats: African giant rats, AP site: apurinic/apyrimidinic site, CNS: central nervous system, DA: dopamine, GFAP: glial fibrillary acidic protein; IgM; immunoglobulin M, iNOS: inducible nitric oxide synthase, MBP: myelin basic protein, MMPs: matrix metalloproteinases, NF-kB: nuclear factor kappa B, NF68: neurofilament 68 protein; TH: tyrosine hydroxylase, V: vanadium. ↑: increase, ↓: decrease.

The present section is focused on the neurotoxic effects of vanadium both in laboratory animals and in those naturally exposed to urban air. Summarized data on this topic, whose main aim is to provide the reader with greater insight into the issues related to the influence of vanadium on CNS, are listed in Figure 11.

As for the influence of environmental pollution with metals on animals living in urban area, Usende et al. [125], who investigated heavy metal pollutants (including vanadium) in selected organs of African giant rats from three agro-ecological zones of Nigeria (i.e., rainforest, woodland/grass savanna, and mangrove/freshwater swamp zones), showed that the concentrations of vanadium in the whole brain of rats from the mangrove/freshwater swamp zone (characterized by increased exploitation of minerals and pipeline vandalization by militants) were higher (by 73% and 71.5%) than those from the rainforest and woodland/grass savanna zones, respectively. These data do not seem to be surprising because vanadium is present in many minerals [11], and the increased exploitation, thereof, can lead to environmental pollution and accumulation in different tissues of animals living in these regions. An elevated concentration of vanadium was also noted by Igado et al. [119] in the olfactory bulb of goats from a relatively unindustrialized area in Nigeria. The results obtained by these authors may in turn suggest that even low scale industrial activities contribute to the accumulation of vanadium in the brain. In turn, studies conducted by Calderón–Garcidueñas et al. [113] showed a higher vanadium concentration in dogs exposed to urban pollution in South West Metropolitan Mexico City. This metal has been detected both in the frontal cortex and in the olfactory bulb, and its level turned out to be higher than the Ni level. Moreover, the hippocampal apurinic/apyrimidinic (AP) sites in the olfactory bulb in the exposed dogs were found to be significantly higher, compared with controls. Additionally, the dogs from the exposed region exhibited neuronal activation of NF-kB, endothelial/glial/neuronal inducible nitric oxide synthase (iNOS) expression, and β-amyloid plaque accumulation. As suggested by the authors, acceleration of AD-type pathology occurred in the dogs chronically exposed to the air pollutants.

An example of findings obtained in studies on laboratory animals is the results reported by Colin-Barenque et al. [118], who analyzed the possible role of matrix metalloproteinases 2 and 9 (MMP-2 and MMP-9) in changes observed in the brain of mice after chronic vanadium inhalation. The authors demonstrated that vanadium increased MMPs in different structures of the CNS and suggested that OS might be involved in the MMP activation. Another study conducted by Colin-Barenque and co-workers [118] that aimed to investigate the relationship between a simple olfactory function test, some enzyme activities, and morphological changes in granule cells from the olfactory bulb as a consequence of vanadium inhalation, demonstrated olfactory impairment at vanadium exposure in a mouse model. The authors observed loss of dendritic spines and necrotic neuronal death in the granule cells of the olfactory bulb [126]. As emphasized, olfactory disorders might be associated with OS generated by this metal. Other researchers examined the neurotoxic properties of vanadium in a mouse model, focusing on the effects of this element on the olfactory bulb to determine whether vanadium subchronic nasal exposure impairs neurobehavioral and neurochemical processes associated with olfactory function. The results revealed a significant decrease in the weight of the olfactory bulb as well as a reduced tyrosine hydroxylase (TH) level and depletion in the dopamine (DA) level in the olfactory bulb. The induction of degeneration processes in the olfactory bulb and in locomotor activities in the vanadium-exposed mice was also observed [120]. In turn, decreased numbers of TH-positive neurons in the substantia nigra and a substantial loss of dendritic spines of the medium-size spiny neurons were noted by Avila–Costa et al. [115], who examined nigrostriatal modifications after vanadium inhalation in a rodent model. Avila–Costa and co-workers [116] also found alterations in the structure of ependymal epithelium characterized by cilia loss, cell sloughing, and ependymal cell layer detachment from the basal membrane. Their studies also showed that vanadium inhaled by mice produced a time dependent loss of dendritic spines, necrotic-like cell death, and notorious alterations of the hippocampus CA1 neuropile, which correlated with spatial memory impairment [117]. Further, Montiel-Flores et al. [112] found a prominent impairment of the cytoskeleton of many nerve cells in rats inhaling vanadium and suggested that inhalation with this metal induces Alzheimer-like cell death. Keil et al. [124], who characterized particulate matter size, metal chemistry, and health effects of dust (including vanadium) collected from the Nellis Dunes Recreation Area (NDRA) located near Las Vegas (NV), found reduced IgM antibody production against neurofilament 68 protein (NF68) and glial fibrillary protein (GEAP) in dust-exposed mice, suggesting that geogenic dust from easily erodible sand dune surfaces at NDRA may have a potential health risk. In turn, a reduced concentration of IgM antibodies against myelin basic protein (MBP) was found by DeWitt et al. [122] in mice exposed to geogenic dust from arsenic-rich sediment (including vanadium) at NDRA. IgM antibodies against MBP were also reduced in mice exposed to geogenic dust (including vanadium) collected from active drainage surfaces (NDRA) [123]. Finally, studies of the levels of vanadium in some internal organs of rodents (in an inhalation model) conducted by Sánchez et al. [114] revealed elevated concentrations of this metal in the brain of mice.

## 6. New Therapies of Neurodegenerative Illnesses—A Summarizing Note

Currently, over 100 substances are being studied to modify the course of AD. More than amyloid and tau protein, it is focused on inflammatory processes. Inflammation is a major sign of aging, and chronic systemic inflammation is associated with reduced brain volume and impaired cognitive function. Although broad-spectrum anti-inflammatory drugs have not improved cognitive outcomes in patients with AD, recent efforts have focused on targeting specific aspects of inflammation that are harmful to the brain while preserving normal immune function. Therapies targeting synaptic plasticity and neuroprotection are also being studied. In addition to these pharmacological approaches, non-pharmacological interventions such as repetitive transcranial magnetic stimulation (rTMS) and transcutaneous direct current stimulation (tDCS), have the potential for clinical application [127]. It is estimated that a global delay in the onset of dementia by 5 years would reduce its incidence by half [128].

As for PD, new therapies focus mainly on disease modification and dopamine resistance symptoms. Some new disease-modifying therapies target α-Syn and its pathways, while others target genes and proteins involved in the pathogenesis of PD, including leucine-rich repeat kinase 2 (LRRK2), parkin and glucocerebrosidase. Great hopes are associated with prasinezumab: an antibody against α-Syn [129]. Disease-modifying pharmacotherapies (such as nilotinib, inosine and isradipine) are being re-examined for the treatment of PD. Antibody therapies, vaccines and immunotherapies aimed at removing abnormal proteins have emerged as promising approaches in preclinical models.

Investigated cellular therapies can be divided into rescue and regenerative therapies; rescue therapy aims to save neurons and slow down disease progression, while regenerative therapy focuses on replacing neurons. Despite these often expressed hopes, these therapies are not intended to cure PD. They are intended to rebuild lost dopaminergic pathway cells without side effects [6]. An alternative symptomatic therapy is adaptive deep brain stimulation (DBS), which involves implanting thin stimulating electrodes into deep parts of the brain. Although it is an invasive procedure, it is characterized by high safety and significant effectiveness in reducing symptoms [130]. Trials are also being conducted using MRI Guided Focused Ultrasound (MRgFUS). Damage of the subthalamic nucleus using MRgFUS can improve quality of life and probably has a beneficial effect on daily activities and the outcome of motor disorder assessment. It is noteworthy that MRgFUS can disrupt the blood–brain barrier, which could find application in the future in pharmacotherapy of Parkinson’s disease, growth factors therapy, nanoparticle therapy or gene therapy [131]. Current efforts to overcome challenges in therapeutic development focus on individualizing therapy and precision in treatment; these principles are particularly important due to the heterogeneity of clinical PD subtypes.

As far as ALS is concerned, the first group of therapies currently being studied is the pharmacological approach. Among the substances that limit inflammation, mastinib stands out. Mastinib is a tyrosine kinase inhibitor on mast cells and macrophages in the central and peripheral nervous system. It had a protective effect on motor neurons through immunomodulation and reducing the inflammatory process in microglia. In patients with ALS receiving mastinib, there was a significantly slower progression of disability. Pegcetacoplan and ibudilast are also undergoing clinical trials. Pegcetacoplan is a synthetic cyclic peptide that binds to components C3 and C3b of the complement system. By blocking these components, it reduces the severity of the inflammatory process. In turn, ibudilast not only inhibits pro-inflammatory cytokines (TNF-α, IL-6) but also matrix metalloproteinase 9 (MMP9), which can potentially accelerate the progression of ALS. It also reduces the activation of astroglial cells, which is associated with the degree of upper motor neuron damage, and has a protective effect on nerve cells. Additionally, it accelerates the removal of TAR DNA-binding protein 43 (TDP-43) and superoxide dismutase 1 (SOD1) protein deposits in cell models with mutations typical for ALS.

There are also some hopes associated with tauroursodeoxycholic acid, which in preclinical studies, reduced OS, endoplasmic reticulum (ER) stress and apoptosis. Currently, its effectiveness is being studied in combination with sodium phenylbutyrate, a histone deacetylase inhibitor that improves transcription and post-transcriptional mechanisms.

Another therapeutic approach that has recently gained renewed interest is the targeting of muscle abnormalities in ALS. The first rationale is neuroprotective, as changes in the muscle and neuromuscular junction may play a role in retrograde degeneration. A second approach is symptomatic by increasing muscle contractility with two troponin activators in development: tirasemtiv and reldesemtiv.

Tofersen is an antisense oligonucleotide whose safety and efficacy have been studied in patients with ALS associated with a pathogenic mutation in the SOD1 gene. The SOD1 protein synthesized on the matrix of the mutated gene acquires additional toxic properties. Inhibiting its synthesis could therefore slow down the progression of the disease.

Many studies have evaluated the usefulness of stem cells in the treatment of ALS. However, no significant effect of treatment on respiratory function or survival time has been reported so far [132].

## 7. Summary, Conclusions, and Further Research Trends

Data summarized in the present review indicate that vanadium, which is able to produce an oxidation-reduction imbalance in the organism, cannot be excluded as a factor playing a role in neurological disorders, in which OS appears to be a part of the pathophysiological mechanism. They also suggest that the level of vanadium in body fluids could be considered as a potential biomarker and predictor of some neurodegenerative syndromes.

At this stage of knowledge, it should not be excluded that vanadium may help in identification of subjects with a higher risk of detrimental effects on CNS. However, more extensive epidemiological studies are needed to provide a conclusive link between pollution with vanadium and neurodegenerative processes in humans.

**Figure 12:**
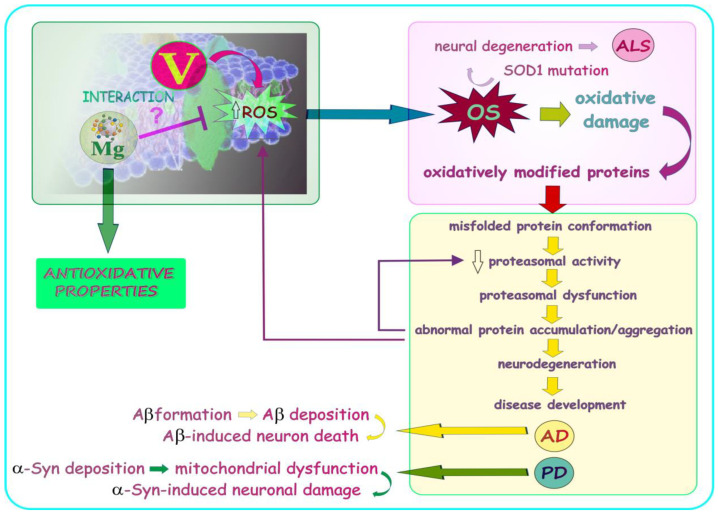
Vanadium in the pathological mechanisms of AD, PD, and ALS. V: vanadium, Mg: magnesium, ROS: reactive oxygen species, OS: oxidative stress, SOD1: superoxide dismutase 1, AD: Alzheimer disease, PD: Parkinson disease, ALS: amyotrophic lateral sclerosis, Aβ: amyloid beta, α-Syn: alpha synuclein. ↓: decrease, ↑: increase.

Finally, the data compiled in the current review clearly indicate that more attention should be paid to the chronic diseases related to vanadium and to the assessment of the environmental vanadium exposure. Additionally, the findings overviewed in this paper may lay the groundwork for further research on the interaction of vanadium with other elements in the context of neurodegenerative processes, especially those with antioxidant potential such as Mg^II^, which has been used in the prevention and therapy of many diseases [133]. Mg^II^ has been reported to be able to limit the pro-oxidative properties of vanadium in the rat liver [134], reduce the degeneration of brain cells, and improve cognitive function [135]. Therefore, a combination of Mg^II^ with vanadium should be considered to be studied in the context of neurological disorders to check if Mg^II^ is able to protect against the harmful effects of ROS and OS in the conditions of vanadium exposure. It is well known that ROS are implicated in vanadium deleterious effects and that OS is an underlying mechanism of vanadium-induced toxicity, as evidenced by the vivo studies [19,136]. Therefore, the examination of the consequences, character, and mechanisms of interactions of vanadium with Mg^II^, as a potential ‘antidote’ limiting vanadium adverse effects and minimizing its strong pro-oxidant properties, is important, and even more crucial in the context of the occupational vanadium exposure and the growing environmental pollution with this metal. Through these kinds of studies, we can find out how important the role of Mg^II^ plays in vanadium intoxication (Figure 12). Moreover, studies on the V^V^xMg^II^ combined effects allow us to better understand the modes of vanadium action and clarify the mechanisms underlying the potential protective role of Mg^II^ in vanadium exposure in mammalian organisms.

As illustrated in Figure 12, OS is implicated in the progression of neurological illnesses, such as AD, PD, and ALS. ROS contributes to the oxidative modification and accumulation of misfolded proteins, which triggers a cascade of events resulting in neuronal damage and neuron death [137,138]. Therefore, detailed investigations targeting evaluations of the consequences and mechanisms of interactions of vanadium with an element having antioxidant potential, including Mg^II^, or with some natural antioxidants during their combined administration, not only addresses a topic of high importance in toxicology, i.e., it refers to the search of approaches limiting vanadium toxicity, but they are also important in the context of possible antioxidant-based therapies for alleviating the severity of neurological diseases.

## Figures and Tables

**Table 1:** Summary of the results of the levels of vanadium in brain and cerebrospinal fluid of patients with neurodegenerative diseases.

Patients	Number	Age (Mean/*Median/Ranges*)	Concentration (Mean/*Median*) *	Biological Fluids/ Tissues	Unit	Ref.
AD cases						
Control	n = 10	88	3.0	FC	ng/g	[57]
AD	n = 14	78	5.0	FC	ng/g	
Control	n = 10	88	<LOQ	VF	ng/mL	[57]
AD	n = 14	78	<LOQ	VF	ng/mL	
Control	n = 54	*73 (60–94)*	*3.2*	CSF	µg/L	[58]
AD	n = 173	*75 (52–86)*	*2.9* ↓	CSF	µg/L	
Control	n = 36	38.5/70.4	ND	CSF	n/a	[59]
AD	n = 33	~76	ND	CSF	n/a	
Control ^†^	n = 30	61 (52–69)	0.0352	Hippocampus	µg/g dw	[60]
AD ^†^	n = 28	63.5 (57–72)	0.0978	Hippocampus	µg/g dw	
Control ^‡^	n = 30	~63 (55–72)	0.0274	Hippocampus	µg/g dw	
AD ^‡^	n = 30	64.5 (61–75)	0.0162 ↓	Hippocampus	µg/g dw	
Control ^†^	n = 30		0.0308	CC	µg/g dw	
AD ^†^	n = 28	61 (52–69)	0.0141 ↓	CC	µg/g dw	
Control ^‡^	n = 30	63.5 (57–72)	0.0272	CC	µg/g dw	
AD ^‡^	n = 30	~63 (55–72)	0.0133 ↓	CC	µg/g dw	
Control ^†,‡^	n = 60	64.5 (61–75)	(0.0117–0.0506)	Hippocampus and CC	µg/g dw	
AD ^†,‡^	n = 58		(0.0086–0.0209)	Hippocampus and CC	µg/g dw	
Control	n = 54	73	3.2 (2.3–5.1)	CSF	µg/L	[61]
AD	n = 174	74	*2.9 (<2.2–5.2)* ↓	CSF	µg/L	
Control	n = 4 (nos = 8)	age-matched	n/a *^f^*	C and PC	ppm	[62]
AD	n = 4 (nos = 8)	78–88	n/a *^f^*	C and PC	ppm	
ALS cases						
Control	n = 10	45 *(26–77)*	*0.09 (0.03–0.19)*	CSF	µg/L	[63]
ALS	n = 17	64 (47–85)	*0.14* ↑ *(0.06–0.77)*	CSF	µg/L	
Control	n = 5	*~56 (43–66)*	0.20	GM	µg/g dw	[64]
ALS	n = 8	~53 (34–66)	0.14	GM	µg/g dw	
Control	n = 5	~56 (43–66)	0.14	WM	µg/g dw	[64]
ALS	n = 8	~53 (34–66)	0.08	WM	µg/g dw	
Control	nos = 3–8	-	0.16	FL (GM)	µg/g dw	[64]
ALS		-	0.15	FL (GM)	µg/g dw	
Control	nos = 3–8	-	0.23	PL (GM)	µg/g dw	[64]
ALS		-	0.13	PL (GM)	µg/g dw	
Control	nos = 3–8	-	0.21	OL (GM)	µg/g dw	[64]
ALS		-	0.09	OL (GM)	µg/g dw	
Control	nos = 3–8	-	0.10	TL (GM)	µg/g dw	[64]
ALS		-	0.18	TL (GM)	µg/g dw	
Control	nos = 3–8	-	0.21	C (GM)	µg/g dw	[64]
ALS		-	0.15	C (GM)	µg/g dw	
Control	nos = 3–8	-	0.26	BG (GM)	µg/g dw	[64]
ALS		-	0.16	BG (GM)	µg/g dw	
Control	nos = 3–8	-	0.12	FL (WM)	µg/g dw	[64]
ALS		-	0.09	FL (WM)	µg/g dw	
Control	nos = 3–8	*-*	0.06	PL (WM)	µg/g dw	[64]
ALS		-	0.07	PL (WM)	µg/g dw	
Control	nos = 3–8	*-*	0.22	OL (WM)	µg/g dw	[64]
ALS		-	0.08	OL (WM)	µg/g dw	
Control	nos = 3–8	-	0.19	TL (WM)	µg/g dw	[64]
ALS		-	0.08	TL (WM)	µg/g dw	
Control	nos = 3–8	-	0.20	C (WM)	µg/g dw	[64]
ALS		-	0.09	C (WM)	µg/g dw	
ALS **	n = 31	*65 (59–70)*	*0.30*	CSF	µg/L	[65]
ALS ^##^	n = 6	*70 (64–82)*	*0.35*	CSF	µg/L	
PD cases						
Control	n = 5	~56 (43–66)	0.20	GM	µg/g dw	[64]
PDC	n = 4	~58 (51–65)	0.21	GM	µg/g dw	
Control	n = 5	~56 (43–66)	0.14	WM	µg/g dw	[64]
PDC	n = 4	~58 (51–65)	0.10	WM	µg/g dw	
Control	nos = 3–8	-	0.16	FL (GM)	µg/g dw	[64]
PDC		-	0.04	FL (GM)	µg/g dw	
Control	nos = 3–8	-	0.23	PL (GM)	µg/g dw	[64]
PDC		-	0.33	PL (GM)	µg/g dw	
Control	nos = 3–8	-	0.21	OL (GM)	µg/g dw	[64]
PDC		-	0.14	OL (GM)	µg/g dw	
Control	nos = 3–8	-	0.10	TL (GM)	µg/g dw	[64]
PDC		-	0.25	TL (GM)	µg/g dw	
Control	nos = 3–8	-	0.21	C (GM)	µg/g dw	[64]
PDC		-	0.15	C (GM)	µg/g dw	
Control	nos = 3–8	-	0.26	BG (GM)	µg/g dw	[64]
PDC		-	0.16	BG (GM)	µg/g dw	
Control	nos = 3–8	-	0.12	FL (WM)	µg/g dw	[64]
PDC		-	0.08	FL (WM)	µg/g dw	
Control	nos = 3–8	-	0.06	PL (WM)	µg/g dw	[64]
PDC		-	0.13	PL (WM)	µg/g dw	
Control	nos = 3–8	-	0.22	OL (WM)	µg/g dw	[64]
PDC		-	0.10	OL (WM)	µg/g dw	
Control	nos = 3–8	-	0.19	TL (WM)	µg/g dw	[64]
PDC		-	0.10	TL (WM)	µg/g dw	
Control	nos = 3–8	-	0.20	C (WM)	µg/g dw	[64]
PDC		-	0.09	C (WM)	µg/g dw	
PD	n = 13	n/a	*0.0372*	CSF	µmol/L	[66]
			1.0541 ↑ (1 wk)	CSF	µmol/L	
			0.2061 ↑ (2 wk)	CSF	µmol/L	
			0.4691 ↑ (4 wk)	CSF	µmol/L	
			0.2944 ↑ (6 wk)	CSF	µmol/L	
			0.1276 ↑ (8 wk)	CSF	µmol/L	
Control	n = 13	~64	0.12	CSF	ng/mL	[67]
PD	n = 26	~65	0.07 ↓	CSF	ng/mL	
Control	n = 12	70	n/a	CN	µg/g dw	[68]
PD	n = 9	73	n/a ^#^	CN	µg/g dw	

* All values are given as reported in the cited reports. n: number of patients; n/a: not available, wk: week. AD: Alzheimer disease, ALS: amyotrophic lateral sclerosis, PDC: parkinsonism-dementia complex, PD: Parkinson disease, FC: frontal cortex, VF: ventricular fluid, CSF: cerebrospinal fluid, LOQ: level of quantitation, GM: gray matter, WM: white matter, FL: frontal lobe, PL: parietal lobe, OL: occipital lobe, TL: temporal lobe, C: cerebellum, CC: cerebral cortex, PC: parietal cortex, BG: basal ganglia, CN: caudate nucleus, DW: dry weight, nos: number of samples. ND: not detectable. ↑ Statistically significant increase. ↓ Statistically significant decrease. ^†^, ^‡^ Eastern Canada and United Kingdom, respectively. ^#^ Detected only in one PD brain (the value for the vanadium level is not provided in the cited report). *^f^* Too low value for the level of vanadium that is difficult to read from the graph provided in the cited report. **, ^##^ ALS patients with a spinal and bulbar onset, respectively.

**Table 2:** Summary of the results of the levels of vanadium in blood, urine, and hair of patients with neurodegenerative diseases.

Patients	Number	Age (Mean/*Median*/Ranges)	Concentration (Mean/*Median)* *	Biological Fluids/Other	Unit	Ref.
AD cases						
Control	n = 54	*73 (60–94)*	*<2.2*	plasma	µg/L	[58]
AD	n = 173	*75 (52–86)*	*<2.2*	plasma	µg/L	
Control	n = 30	74	0.0613	serum	µg/L	[69]
AD	n = 30	~80	0.0673	serum	µg/L	
Control	n = 40	65 (57–87)	0.04 (0.03–0.09)	serum	µg/L	[70]
AD	n = 34	72 (54–84)	0.08 ↑ (0.03–0.53)	serum	µg/L	
Control	n = 17	~74	0.17	hair	µg/g	[71]
AD	n = 9	~83	0.06	hair	µg/g	
Control	n = 18	age-matching	39.9	serum	ng/g	[72]
AD	n = 30	60 (45–84)	76.3 ↑	serum	ng/g	
Control	n = 161	>60	n/a ^#^	plasma	n/a	[73]
AD	n = 92	>60	n/a ^#^ ↑	plasma	n/a	
Control	n = 124	44.8 (20–84)	*0.06 (0.05–0.08)*	serum	µg/L	[74]
AD	n = 53	74.5 (58–86)	*0.04 (0.03–0.07)*	serum	µg/L	
Control	n = 44	>45	*0.06*	serum	ng/mL	[75]
AD	n = 60	74.6	0.05	serum	ng/mL	
Control	n = 44	>45	0.09	WB	ng/mL	[75]
AD	n = 60	74.6	0.13	WB	ng/mL	
PD cases						
Control	n = 124	44.8 (20–84)	*0.06 (0.05–0.08)*	serum	µg/L	[74]
PD	n = 71	65.5 (41–81)	*0.11 (0.06–0.16)*	serum	µg/L	
Control	n = 44	~52	0.06	serum	ng/mL	[76]
PD	n = 71	65.5	0.11 ↑	serum	ng/mL	
Control	n = 13	~64	0.07	WB	ng/mL	[67]
PD	n = 26	~65	0.22 ↑	WB	ng/mL	
Control	n = 13	~64	0.15	serum	ng/mL	[67]
PD	n = 26	~65	0.17	serum	ng/mL	
Control	n = 13	~64	0.10	urine	ng/mL	[67]
PD	n = 26	~65	0.20 ↑	urine	ng/mL	
ALS cases						
Control	n = 9	45 *(26–77)*	*0.07*	plasma	µg/L	[63]
ALS	n = 15	64 (47–85)	*0.07*	plasma	µg/L	
Control	n = 100	51	0.08	hair	µg/g	[77]
ALS	n = 100	55	0.05	hair	µg/g	

* All values are provided as reported in the cited reports. n: number of patients; n/a: not available. AD: Alzheimer disease, PD: Parkinson disease, ALS: amyotrophic lateral sclerosis, WB: whole blood, ↑ Statistically significant increase. ^#^ The values for the level of vanadium have not been provided by the authors. ↑ Higher level but not statistically significant, compared to the control.

**Table 3:** Summary of the neurological side effects and neurobehavioral changes as well as the levels of vanadium in body fluids of humans exposed to vanadium occupationally, subjects with acute vanadium poisoning and the elderly.

Item	Number	Age (Mean/Ranges)	Concentration (Mean/Range) *	Body Fluids	Unit	Findings	Ref.
Occupationally exposed							
WEV	n = 49	41.4 (20.1–57.1)	7.5 (2.18–46.4)	serum	µg/L	↓ attention	[78]
			C: 0.8 (0.3–3.1)			↓ visuospatial abilities	
	n = 49	41.4 (20.1–57.1)	14.4 (2.12–95.4)	urine	µg/L		
			C: 0.4 (0.07–1.4)				
WEV	n = 19 (2)	~34	15.5 (3.0–35.2)	urine	µg/L	tremor of finger	[79]
			C: 6.0				
WEV	n = 463	39.5 (20–60)	n/a	n/a	n/a	↑ anger-hostility	[80]
						↑ depression-dejection	
						↑ fatigue-inertia	
						↓ vigor-activity	
						↓ auditory/visual memory	
						↓ perception/motion speed	
						↓ coordination	
WEV	n = 106	n/a	n/a	n/a	n/a	↙ vision-memory	[81]
						↙ motor speed and accuracy	
						mood disorders (↗ negative moods)	
WEV	n = 128	n/a	n/a	n/a	n/a	↙ hearing and visual memory	[82]
						↙ movement, velocity, accuracy	
						↙ coordination	
Acute poisoning cases							
Woman	n = 1	22	61.5 ^†^/12.3 ^††^ <20	urine CSF	µg/L µg/L	right-sided brachiofacial paresis right hemihypesthesia right sensomotor hemiparesis mixed amnestic and sensorimotor aphasia	[83]
Elderly subjects							
Male/female	n = 50	≥60	24.44 (6.0–63.0)	WB	µg/L	↓ general cognitive function	[22]
			Y: 22.8 (6.0–49.0)	WB	µg/L		

* All values are given as reported in the cited reports. n: number of people; n/a: not available. WEV: workers exposed to vanadium, C: control group, Y: young, CSF: cerebrospinal fluid, WB: whole blood. ↓: statistically significant reduction, ↑: statistically significant increase, ↙: decrease. ↗: increase. ^†^, ^††^ after 3 and 9 months, respectively. Numbers in squared brackets indicate the number of people with symptoms.

## Data Availability

Data sharing not applicable.
